# Making Sense of Composite Endpoints in Clinical Research

**DOI:** 10.3390/jcm12134371

**Published:** 2023-06-29

**Authors:** Daniela Baracaldo-Santamaría, John Edwin Feliciano-Alfonso, Raul Ramirez-Grueso, Luis Carlos Rojas-Rodríguez, Camilo Alberto Dominguez-Dominguez, Carlos Alberto Calderon-Ospina

**Affiliations:** 1 Pharmacology Unit, Department of Biomedical Sciences, School of Medicine and Health Sciences, Universidad del Rosario, Bogotá 111221, Colombia; daniela.baracaldo@urosario.edu.co (D.B.-S.); raul.ramirez@urosario.edu.co (R.R.-G.); luisca.rojas@urosario.edu.co (L.C.R.-R.); 2 School of Medicine, Universidad El Bosque, Bogotá 110121, Colombia; jfeliciano@unbosque.edu.co; 3 School of Medicine and Health Sciences, Universidad del Rosario, Bogotá 111221, Colombia; camilo.dominguez@urosario.edu.co; 4 Research Group in Applied Biomedical Sciences (UR Biomed), School of Medicine and Health Sciences, Universidad del Rosario, Bogotá 111221, Colombia

**Keywords:** randomized controlled trials, data interpretation, treatment outcome, outcome assessment, endpoint determination

## Abstract

Multiple drugs currently used in clinical practice have been approved by regulatory agencies based on studies that utilize composite endpoints. Composite endpoints are appealing because they reduce sample size requirements, follow-up periods, and costs. However, interpreting composite endpoints can be challenging, and their misuse is not uncommon. Incorrect interpretation of composite outcomes can lead to misleading conclusions that impact patient care. To correctly interpret composite outcomes, several important questions should be considered. Are the individual components of the composite outcome equally important to patients? Did the more and less important endpoints occur with similar frequency? Do the component endpoints exhibit similar relative risk reductions? If these questions receive affirmative answers, the use and interpretation of the composite endpoint would be appropriate. However, if any component of the composite endpoint fails to satisfy the aforementioned criteria, interpretation can become difficult, necessitating additional steps. Regulatory agencies acknowledge these challenges and have specific considerations when approving drugs based on studies employing composite endpoints. In conclusion, composite endpoints are valuable tools for evaluating the efficacy and net clinical benefit of interventions; however, cautious interpretation is advised.

## 1. Introduction

A composite endpoint (CEP) is an outcome that combines two or more endpoints of interest within a single variable [1,2]. In this way, patients are said to have achieved the clinical outcome if they suffer from any one of the events from the predetermined composite components. These types of outcomes emerged primarily to reduce the necessary sample size and follow-up period, while preserving the statistical power to evaluate the efficacy and safety of new therapies [3]. Most CEPs are analyzed from the time of randomization of the patient to the first occurrence of any of the components of the composite, as a time-to-first-event analysis. While they are widely used in medicine and economics, it is rare to encounter this type of measurement in other sciences. In medicine, CEPs are commonly used to evaluate outcomes in clinical trials, but also to assess the performance of health systems in cost-effectiveness analysis (e.g., quality-adjusted life years or QALYs, and disability-adjusted life years or DALYs) [4]. 

The use of CEPs as primary outcome measures in randomized clinical trials (RCTs) is especially common in cardiology and oncology, where they play a major role in providing information about the net effect of an intervention [5]. For instance, the most commonly used CEP in cardiovascular clinical trials is termed major adverse cardiac events (MACE), which usually includes a composite of nonfatal stroke, nonfatal MI (myocardial infarction), and cardiovascular death [6]. MACE as an endpoint is usually tailored to the study population, and the components of the composite can vary to adjust better to the study’s purpose, for instance replacing stroke with stent thrombosis or revascularization. Although the most employed variables in CEPs are clinically important (hard) outcomes, there are also trials with endpoints that incorporate surrogate variables in the CEP. For instance, when some clinical trials include disease progression as part of the CEP, surrogate variables are often used to determine this outcome. As an example, a decrease in the 6 min walk distance is used in pulmonary arterial hypertension trials to measure disease progression [7]. Regulatory agencies usually require the surrogate outcome to be previously validated in epidemiological studies and clinical trials to ensure that the surrogate endpoint is reliable to predict or correlates with clinical benefit [8]. 

This review aims to provide a guide on how to correctly interpret CEPs, to highlight common challenges encountered during their interpretation and provide practical strategies to assist physicians in making informed clinical decisions when reading studies that employ composite endpoints as primary outcomes. The initial three sections primarily target clinicians, while the last two sections cater to a broader range of stakeholders involved in the utilization of CEPs, such as methodologists and statisticians.

## 2. Unveiling the Advantages and Limitations of Composite Outcomes: Representative Examples

The importance of correctly using and interpreting CEPs lies in their influence on clinical decisions. For example, the current European guidelines for the treatment of ST segment elevation myocardial infarction (STEMI) and non-ST segment elevation myocardial infarction (NSTEMI) [9,10] recommend the use of ticagrelor over clopidogrel based on the results of the PLATO (Platelet Inhibition and Patient Outcomes) study. This study was a multicenter, randomized, double-blind clinical trial [11] comparing ticagrelor and clopidogrel for the prevention of cardiovascular events in patients with acute coronary syndrome (ACS). The primary endpoint was a composite of death from vascular causes, MI, or stroke. The group that received ticagrelor had an occurrence of the composite outcome of 9.8% compared with 11.7% of the group receiving clopidogrel (hazard ratio 0.84; 95% confidence interval [CI] 0.77 to 0.92; *p* < 0.001) [11]. Based on these results, treatment with ticagrelor compared to clopidogrel reduced the composite outcome. In order to better analyze and interpret these types of results, we would need to see if the components of the composite endpoint are of similar importance, if they occur with similar frequencies, and if they share similar relative risk reductions [12]. In this case, the use of the composite outcomes fulfilled these criteria, and the use of ticagrelor is now preferred to that of clopidogrel in some cases. 

Not only are composite outcomes widely used in cardiology, but also there have been important milestones in clinical research with composite outcomes in other areas (Table 1). In oncology, a commonly used CEP to evaluate efficacy is progression-free survival (PFS), which includes death and disease progression as the components of the composite, defined as the time from the start of the treatment to either progression or death from any cause [13,14]. This CEP is used in oncology to decrease the sample size and the follow-up period it would normally take to measure the overall survival (OS) and it is also included as a suitable surrogate endpoint for regulatory approval of cancer drugs [15].

For instance, pembrolizumab, a PD-1 (programmed cell death protein 1) receptor-blocking antibody, has recently been approved by the FDA as a first-line therapy for the treatment of MSI-H/dMMR (microsatellite instability-high/mismatch repair deficient) colorectal cancer [16]. Approval was based on the KEYNOTE trial (NCT02563002), a phase 3, open-label trial comparing the efficacy of pembrolizumab to that of standard chemotherapy. The primary efficacy outcomes were PFS and OS. Treatment with pembrolizumab showed superior outcomes compared to chemotherapy in terms of PFS, measured by the Response Evaluation Criteria in Solid Tumors (RECIST), or death from any cause [17]. Although the composite of death and disease progression can be useful to evaluate efficacy, the use of PFS in oncology has been widely criticized [18,19,20], mainly because PFS does not necessarily mean an increased quality of life. Therefore, it is not only important to adequately interpret and analyze CEPs, but one also needs to understand their limitations according to the context in which they are being used. 

**Table 1:** Examples of trials incorporating primary CEPs that had an impact on patient care.

Area	Trial	Composite Outcome	Interpretation	Impact on Patient Care	Reference
Cardiovascular	PLATO	MACE: death from vascular causes, MI, or stroke	Treatment with ticagrelor compared with clopidogrel reduced the rate of the composite endpoint	-Current guidelines for the treatment of NSTEMI and STEMI recommend the use of ticagrelor over clopidogrel	[9,10,11]
	TRITON-TIMI-38	MACE: death from cardiovascular causes, nonfatal MI, or nonfatal stroke	Treatment with prasugrel compared with clopidogrel reduced the rate of the composite endpoint	-Current guidelines for the treatment of NSTEMI and STEMI recommend the use of prasugrel over clopidogrel	[9,10,21]
	PARADIGM-HF trial	Death from cardiovascular causes or a first hospitalization for heart failure	Treatment with sacubitril–valsartan versus enalapril in patients with heart failure significantly reduced the rate of the composite outcome	-Served as the basis to justify the regulatory approval and the clinical use of sacubitril–valsartan for the treatment of heart failure	[22]
Oncology	KEYNOTE	Progression-free survival (considered as the time from randomization to first disease progression according to RECIST, or death from any cause)	Significant improvement in PFS for patients randomized to pembrolizumab compared with chemotherapy	In patients with non-operable metastatic colorectal cancer with deficient DNA mismatch repair/microsatellite-unstable tumors, the use of pembrolizumab as monotherapy is recommended as a first-line treatment option over cytotoxic chemotherapy	[16,17,23]
Nephrology	DAPA-CKD	≥50% reduction in eGFR, end-stage kidney disease, and death from a kidney or cardiovascular cause	Treatment with dapagliflozin reduced the hazard of the primary composite	Dapagliflozin was approved for the indication to reduce the risk of kidney function decline, end-stage kidney disease, cardiovascular death, and hospitalization for heart failure in adults with CKD at risk of progression	[24,25]

RECIST: Response Evaluation Criteria In Solid Tumors, HIF-PHI: hypoxia-inducible factor-prolyl hydroxylase inhibitors.

Like most variables, CEPs have some advantages and disadvantages [26] (Table 2). Having shorter follow-up periods may offer a significant advantage, as it enables researchers to obtain results in a shorter time frame. This can facilitate earlier access to treatments, particularly for medical conditions with unmet needs, while also allowing for early initiation of post-marketing surveillance. A systematic review that analyzed the rationale, the interpretation, the advantages, and the limitations of CEPs used in publications between the years 1980 and 2005 found that the decrease in sample size requirements and the ability to assess the net effect of an intervention were the most commonly cited advantages [27]. Furthermore, the authors concluded that researchers paid little attention to the methodological basis for using and interpreting CEPs and that their views were often contradictory. Consequently, when choosing the primary outcome of a clinical trial, researchers might prefer to use a CEP to decrease the necessary sample size and follow-up period but also to reduce costs. Nonetheless, CEPs can often be misleading, and this is one of the major critiques of them. For instance, a systematic review published in 2008 that analyzed how CEPs were defined, reported, and interpreted in clinical trials found that in the majority of studies, the components were unreasonably combined, not well defined, and reported inadequately [28], sometimes leading to an exaggerated perception of the effects of an intervention or to confusing results. Similarly, another systematic review analyzed the use of CEPs in cardiovascular clinical trials between the years 2002 and 2003 and found that there was often a large to moderate gradient among the components of the composite in terms of importance to patients and the magnitude effect, that could lead to misleading conclusions [29].

## 3. A Concise Guide to Interpreting Composite Outcomes in Clinical Practice

An example where the use of CEPs can confuse readers and lead to a difficult interpretation of the results is in the trial by Kastrati et al. [30]. This randomized double-blind trial aimed to compare bivalirudin with unfractionated heparin in patients with stable or unstable angina who were undergoing percutaneous coronary intervention (PCI) with stent placement and who previously received pretreatment with clopidogrel. The primary outcome was a CEP of death from any cause, MI, urgent revascularization due to myocardial ischemia within 30 days after randomization, or major bleeding. When we take a look at the results (Table 3), they can be confusing because we can see that different drugs were favored differently according to the component of the CEP we analyze [30]. For instance, when looking at the incidence of MI, the group that received unfractionated heparin had fewer events, which would favor the use of unfractionated heparin; however, when looking at major bleeding, we can clearly see that the patients receiving bivalirudin had fewer events. This is sometimes difficult to interpret and is a reason why CEPs can be confusing sometimes. However, when analyzing the CEP with the four components together, we see that bivalirudin did not significantly reduce the overall incidence of the CEP. This is one of the reasons why the current indication to use bivalirudin in patients undergoing PCI is for those with, or at risk of, heparin-induced thrombocytopenia or heparin-induced thrombocytopenia and thrombosis syndrome [31].

As a response to this problem, certain “rules” have been described by Montori et al. [12] for the correct interpretation of CEPs and for the validation of these types of outcomes.

The first is that the constituents of the composite should be of similar importance to patients [32]. Ideally, if all the components of the composite were of equal importance to patients, then it would not be misleading to assume that the effect of the intervention is comparable on each component of the composite. This means that even if the effects of the treatment vary on the different components of the composite, a clinical net effect of the intervention will not have a negative impact on decision making. However, this is not always the case, and the components are sometimes unreasonably combined [33]. For instance, taking the example discussed by Palileo-Villanueva et al. [32], the DREAM trial (Diabetes Reduction Assessment with ramipril and rosiglitazone Medication) [34] aimed to evaluate rosiglitazone’s ability to prevent type 2 diabetes in individuals at high risk; however, they used as a primary outcome the composite of incident diabetes or death. These two individual outcomes are evidently of very different importance to patients. The results of this RCT showed 306 (11.6%) individuals given rosiglitazone and 686 (26.0%) given placebo developed the primary outcome [34]. One could incorrectly interpret that rosiglitazone decreased mortality when in fact, when looking closely at the effect of the intervention in the components, it did not (Table 4). 

Therefore, when interpreting CEPs in a clinical trial, the following question should be answered: are the component endpoints of the CEP of similar importance to patients [12]? If the answer is no, the results could be incorrectly interpreted, and further investigation of the results is needed. For instance, looking at the results of the CRISTAL randomized trial [35], the investigators aimed to determine whether aspirin was noninferior to enoxaparin in preventing venous thromboembolism (VTE) after total hip arthroplasty or total knee arthroplasty. The primary outcome was a CEP of symptomatic VTE including pulmonary embolism and deep venous thrombosis above or below the knee. The study failed to demonstrate the non-inferiority of aspirin compared to enoxaparin, and the authors concluded that patients receiving aspirin compared to enoxaparin had a higher rate of symptomatic VTE within 90 days after total hip arthroplasty or total knee arthroplasty [35]. The results are presented in Table 5. One could argue that pulmonary embolism and proximal (above the knee) deep vein thrombosis have a greater impact on the patient than distal (below the knee) thrombosis, and therefore the use of the composite outcome in this case is not in compliance with the first rule [36,37]. This demonstrates that when reading clinical trials with CEPs as primary endpoints, it is very important to see which components of the composite are driving most of the results. We can see in Table 5 that the difference between aspirin and enoxaparin in VTE rates was mostly due to the differences in the rates of below-the-knee VTE, which might be the least important outcome for the patient. This example highlights the importance of selecting the components of the CEP properly. 

This leads us to the second key question to consider when interpreting composite outcomes: did the more and less important endpoints occur with similar frequency [12]? If the occurrence of the components is not similar, then the CEP will be mostly determined by the predominant event [32]. Taking the example from the CRISTAL trial [35] (Table 5), we can see that deep venous thrombosis below the knee was the component of the composite with most events (129), largely driving the difference between the treatments. Furthermore, this is also evident in the results of the DREAM trial [34] (Table 4), where the most frequent outcome was the development of diabetes compared to death. Therefore, the reduction in the CEP was mostly driven by the reduction in diabetes. The problem is that if the more important outcomes occur less frequently than the less important ones, the CEP becomes misleading [12].

The third key question is: can one be confident that the component endpoints share similar relative risk reductions [12]? This final question refers to the effect of the treatment on each component of the composite. Montori et al. pointed out that the biology of the components of the composite should be similar, such that we would expect similar risk reductions across all the components [12]. For instance, in the DREAM trial it is highly plausible that treatment with rosiglitazone could reduce the event of “incident diabetes”; however, the other component of the composite was death, which could be the result of many contributors. 

In addition to looking at the biological rationale of the CEP, the relative risk reductions should be observed for each component to see if the treatment effect was indeed similar in all components. As an example, in the LIFE trial (Losartan Intervention For Endpoint reduction in hypertension) [38], the investigators aimed to establish if losartan, compared to the standard of care at that time (atenolol), could reduce the primary CEP. The CEP components were cardiovascular mortality, stroke, and MI. These are reasonable single endpoints to assemble a CEP and have a strong biological rationale [39]. The authors concluded that losartan prevented more cardiovascular morbidity and death than atenolol in patients with hypertension [38]. The results of the trial are presented in Table 6. 

When taking a closer look at the results, we can see that the conclusion of the study could be misleading. Both the confidence interval for the adjusted hazard ratio for cardiovascular mortality and MI contain 1; therefore, it would be wrong to conclude that losartan reduced either outcome. Furthermore, the losartan-treated group even had an increased risk for MI compared to atenolol, although not statistically significant. The LIFE trial suggests that losartan may reduce the risk for stroke, but it has unclear effects on the other two components of the CEP.

On the other hand, there are some cases where CEPs fulfill the three parameters previously discussed. For example, Webster et al. [40] conducted a randomized controlled trial to compare the routine replacement of IV peripheral catheters (usually every 72 h) compared to only replacing them when clinically indicated, to measure catheter failure and the associated costs. The authors used a CEP as the main outcome that included phlebitis or infiltration. First, we can see these two components of the composite have similar importance to the patients, because the occurrence of either of them means the catheter has failed and a replacement is necessary. To analyze the following questions, we will need to take a closer look at the results (Table 7) [40]. When considering if the more and less important endpoints occur with similar frequency, we can see that there are more cases of infiltration than of phlebitis; however, infiltration in this case, as the authors well explained in the paper, may suggest an underlying phlebitis process in the vein wall. Furthermore, we would expect to see similar risk reductions in both components of the composite because they share similar pathophysiological processes. Therefore, the use of this CEP would be correct according to the three questions we previously established, and it is also noteworthy that the authors clearly explained the rationale for using the composite measure, which in this case was to avoid any potential misdiagnosis [40]. 

Another good example to elucidate the correct use of CEPs is the HOPE (Heart Outcomes Prevention Evaluation) study [41]. This trial was a double-blind randomized placebo-controlled trial to study the effects of ramipril in patients with high risk of presenting cardiovascular events vs. placebo, using a CEP of MI, stroke, or death from cardiovascular causes. The results showed that 14% (n = 651) of the patients treated with ramipril experienced the primary outcome compared to 17.8% in the placebo arm (n = 826) [41]. Regarding the importance of the components to the patient, it is reasonable to combine death from cardiovascular causes with other disabling conditions such as MI or stroke. Furthermore, when looking at the results (Table 8), we can see that the effect of the intervention was relatively homogeneous, with a relative risk ranging from 0.7 to 0.8 in all the components of the composite. In addition, the placebo-treated group had worse outcomes with a statistically significant difference compared to the ramipril group in all components and the CEP. It would therefore be correct to conclude that the effect of the intervention was positive for all the components of the CEP. The interpretation of this CEP is easy because it leaves no space for conclusions that might be misleading. 

The questions addressed in this section provide a practical approach that is highly valuable for clinicians in accurately interpreting CEPs in clinical studies. Table 9 summarizes these questions. 

## 4. What Is the Point of View of Regulatory Agencies?

Both the FDA (Food and Drug Administration) and the EMA (European Medicines Agency) have guidelines on how to handle CEPs. 

When handling CEPs from a regulatory point of view, the EMA also describes the three “rules” for the validity of the CEP that we discussed in Section 2 with some special considerations [3]. The first mentions that when using a CEP as a primary outcome, it should meet the same requirements as a single primary endpoint: that is, being capable of providing the key evidence of efficacy required for a license. The second consideration is that the treatment should be expected to affect all components in a similar way, which reminds us of the third key question discussed in Section 2: whether one can be confident that the component endpoints share similar relative risk reductions. The components of the composite must make sense from a clinical perspective, and the assumption that the treatment effect will be similar in all components should be possible and should be based on past studies of a similar type [3]. 

Furthermore, from a regulatory point of view, it is especially relevant when one or more of the components is adversely affected by the treatment but is masked by the CEP (by a large beneficial effect on the other components), which leads us to the third consideration: the clinically more important components should at least not be affected negatively [3,12]. This is why the EMA encourages that single components should be analyzed separately to provide supportive information [3]. In this way, we can see if the treatment does not benefit all components of the composite, especially the more important ones [3]. This is especially relevant when the components of the composite are of high clinical importance, for example, all-cause mortality as a part of the composite should always require a separate analysis, which should be considered at the study protocol planning stage. In addition, this analysis is also necessary to account for competitive risks, for instance, when a CEP has death in the components, it means the patients that die are no longer at risk of presenting an additional outcome of the CEP, thereby influencing the results [42]. 

The EMA specifies that if the effect of the treatment on one of the components of the composite is in the product information, it should be clearly supported by the data, implying that each component must be analyzed separately. In this context, claims about the treatment effect on the component that had the lowest frequency in the composite are carefully phrased. For example, in the DREAM trial, a claim that rosiglitazone decreased mortality would lack evidence and is therefore not incorporated in the product information [3]. Furthermore, the EMA has launched a new system called The Clinical Trials Regulation, whereby first the sponsors of the clinical trial have to submit a dossier to the national competent authority (depending on the country the clinical trial is going to be held) and then they have to submit an online application for approval to run a clinical trial in a European country. This increases the transparency of information on clinical trials so that the regulatory agency can evaluate the design and results of the trial more closely [43]. 

The FDA also agrees with the points mentioned in Section 2 [44] and highlights the components should be of similar importance, otherwise the CEP will not be a reasonable indicator of the drug’s benefit. They additionally emphasize that the components of the composite should be individually examined and should always be included in the results. Regarding considerations of the statistical analysis, they clearly specify that if the individual components of the composite are to be analyzed separately, this should be prespecified in the statistical analysis plan and should account for multiplicity. Furthermore, the FDA states that in order to demonstrate an effect on an individual component of the composite, it should be included prospectively as a secondary outcome or even as an additional primary endpoint to avoid misleading conclusions. 

As far as we know, there is no explicit regulation regarding the use of CEPs in clinical research by the most important Latin American drug regulatory agencies.

As a final observation, the role of regulatory agencies is to ensure that CEPs meet their criteria to be considered valid. They also have the responsibility of verifying that the individual components make sense and carry similar implications for the patient. When a clinical trial achieves statistical significance, this does not necessarily imply regulatory approval. This is where the role of the regulatory agencies is most important, as it validates the use of the CEP and provides a specific label based on the analysis of the results. 

## 5. New Perspectives for the Statistical Analysis of Composite Endpoints

One of the major critiques of composite outcomes is the way in which they are analyzed. There are two ways in which CEPs can be analyzed, depending on whether the components of the CEP correspond to distinct events (that is, the individual events within the composite outcome are considered separate events with their own clinical significance and the occurrence of any one of these events would contribute to the composite endpoint) or not. If the components correspond to distinct events, then they are usually assessed as time to first occurrence where the event is any one of the components. However, in some situations the patients might experience more than one event, in which case it is appropriate to analyze the total endpoint events. In the first case, the CEP is analyzed by time-to-first-event analysis, where a cox model, a log-rank test, and Kaplan–Meier plots are used and produce a hazard ratio, a confidence interval, and a *p*-value [44,45]. In the second case, all events are considered irrespective of their order of occurrence, and each patient can be included in the event counts for more than one component. Statistical approaches that account for all events are the negative binomial regression, the Andersen–Gill regression, and the weighted CEP analysis, which will be briefly discussed in the next paragraphs [46]. Nevertheless, when using this approach, the analysis can be complicated by competing risks, where the occurrence of some endpoints (e.g., death) make it impossible for the other events to occur in the same patient, causing one group to appear to have a more likely profile with respect to other endpoints [44].

Time-to-first-event analysis has been the usual method for analyzing these types of outcomes, although it has been widely criticized because it assumes that the components of the endpoints have equal severity and only takes into account the first endpoint. For instance, if a patient presented with a non-fatal MI and then subsequently died, only the non-fatal MI would be considered. To overcome these limitations, some statistical methods have emerged: some of them consider all events for each patient, as mentioned before, while others take event severity into account by assigning weights to each component [46]. 

Furthermore, one of the novel statistical approaches to overcome the limitations of the time-to-first-event analysis is the win ratio analysis first described by Pocock et al. [45]. With this method, the components of the composite are first ranked by their severity (e.g., in MACE, the most severe component will be death, followed by stroke, and then by MI). Secondly, the patients on each arm of the clinical trial (receiving the new drug vs. standard care or placebo) are paired up according to their individual risk estimates, and then each pair is evaluated to see which patient had the most severe event. If none of the patients had the most severe event (usually death), then the next severe component according to the previous established rank (e.g., stroke) is evaluated, and so on [46]. In this way, winner and loser pairs are determined, or in the case that neither of the patients presented an event, the pair is said to be tied. Pairs that win are those in which the control group experienced the event first. Finally, the win ratio is calculated by the number of winners divided by the number of losers. Then, the 95% confidence interval and *p*-value for the win ratio are computed [45]. Deep statistical considerations of this analysis are beyond the scope of this review. For more detailed information, readers can refer to the aforementioned article [45].

Time-to-first-event analysis is also criticized because the subsequent events that occur following the first event are lost in the composite [47]. The analysis of these “lost“ events is important to evaluate the patient’s quality of life, medical costs, and efficacy in some cases. For example, it has been proposed that the evaluation of repeated non-fatal events along with fatal events may be more accurate to assess the true burden of heart failure and the impact of the treatment. In heart failure, hospitalization is still a common problem, and readmission represents a major part of the overall cost of the illness [48,49]; therefore, it would not be appropriate to use a time-to-event analysis. A way to overcome this limitation is by evaluating all events between the two groups and then comparing the number of events. This approach is mostly used in clinical trials that want to assess recurrent events while using a CEP as a primary outcome. The PARADIGM-HF trial [48], for example, used a CEP of cardiovascular death or hospitalization for heart failure as a primary outcome to evaluate the effect of sacubitril/valsartan vs. enalapril on recurrent events, including all hospitalizations. In such cases, the distribution of clinical events is represented by an overdispersed Poisson distribution, and therefore the statistical method used is a negative binomial regression. When negative binomial regression analysis is not possible, for example because the patients have different follow-up periods, then researchers can use the Andersen–Gill model, which is an extension of the traditional Cox model. Additional statistical considerations can be consulted in the following reviews [46,50]. 

Another useful statistical analysis that has appeared over the last decade to analyze CEPs is the Weighted Composite Endpoint analysis [51]. This method is an extension of the time-to-event method in which a weight is assigned to each non-fatal event while incorporating all events into the analysis. For example, in the case of MACE, authors assign death a weight of 1.0, while stroke and MI might receive weights of 0.47 and 0.38, respectively [52,53]. The weight scale for the outcomes is usually derived from a Delphi panel (a panel of experts that reach a consensus after multiple rounds of questionnaires). At the start of the analysis, each patient starts with a residual weight of 1.0, which remains the same if no event occurs until the end of the follow-up. The events reduce a patient’s residual weight by the weight previously assigned to each event, and this can be used to create a modified life table with a weighted number of patients at risk [46].

A good way to understand the impact of these statistical approaches in the analysis of a clinical trial is by looking at the work carried out by Hara et al. [54]. They compared the use of different statistical approaches (win ratio, negative binomial regression, Andersen–Gill analyses, and weighted composite endpoint analysis) to analyze the results of the GLOBAL LEADERS trial [55]. The trial was a prospective, randomized, open-label, multicenter, superiority trial. It aimed to compare the benefits and risks of ticagrelor as monotherapy to conventional dual antiplatelet therapy in the population undergoing PCI, using a composite primary outcome of all-cause mortality or new Q-wave MI. The study was originally assessed using a time-to-first-event analysis, and the results showed no superiority of ticagrelor over conventional dual antiplatelet therapy. In the analysis by Hara et al. [54], the composite outcome included mortality for all causes, revascularization, any reported stroke, MI, or bleeding events. Using the traditional time-to-first-event analysis, the HR was 0.94 (95% CI 0.88–1.01; log-rank *p* = 0.10); when using the win ratio analysis, the win ratio was 1.05 (95% CI 0.97–1.13; *p* = 0.20); by analyzing recurrent events with negative binomial regression (rate ratio 0.92 [95% CI, 0.85–0.99; *p* = 0.020]) and Andersen–Gill analyses (hazard ratio 0.92 [95% CI, 0.85–0.99; *p* = 0.028]), ticagrelor monotherapy showed significant statistical advantage, while when using the weighted composite endpoint analysis, the hazard ratio was 0.93 (95% CI, 0.84–1.04; log-rank *p* = 0.22) [54]. This study shows the importance of applying multiple statistical methods to obtain a more accurate and useful analysis. The advantages and limitations of the novel statistical approaches are described in Table 10.

## 6. Conclusions

CEPs are widely used and will continue to be used because they provide many statistical and economic advantages, while facilitating the access to new therapies in shorter periods of time. The use of CEPs has been criticized because they can sometimes be misleading, and in some cases the conclusions drawn by the authors are ambiguous. It is important that professionals in the health care system (including the regulatory agencies) know how to interpret these results correctly and to analyze if the variables included in the CEPs make sense before making a clinical decision. The international regulatory agencies (EMA and FDA) are aware that the results can be confusing if the CEPs are not correctly presented and analyzed; so, they explicitly ask for a report on the individual components of the composite. Furthermore, they propose some requisites that the CEPs must follow that are in accordance with the method for assessing the validity of CEPs described by Montori et al. It should also be noted that the indications and uses of the products are determined based on the analysis performed by the regulatory agencies. In addition, when considering the statistical analysis of the CEPs, the traditional time-to-first-event analysis has many limitations and may not reflect all the facets of a trial. Multiple statistical methods for the analysis of these types of outcomes should be used as, together, they may yield a more accurate and appropriate analysis. Despite their complexity, CEPs are an important tool for evaluating the efficacy and net clinical benefit of interventions in clinical trials, have provided valuable information that has led to the approval of many drugs, and have proven to be a useful tool when used correctly.

## Figures and Tables

**Table 2:** Advantages and disadvantages of CEPs.

Advantages	Disadvantages
Reduced sample size requirement Reduced follow-up period Reduced costs Can assess the net clinical benefit of an intervention (it can capture the trade-off between benefit and harm by including variables of safety and efficacy)	They can be difficult to interpret Frequently, the components of the composite outcome are not explicitly discriminated Authors generally do not provide a complete discussion regarding the scope of the results of the CEP They do not distinguish the relative clinical significance of each component (if analyzed traditionally *) Can lead to misleading conclusions Counts only the first occurrence of any event (if analyzed traditionally *)

* New statistical methods have emerged to overcome these issues (see Section 4).

**Table 3:** Results of the trial “Bivalirudin versus Unfractionated Heparin during Percutaneous Coronary Intervention”.

Endpoint	Bivalirudin Group (n = 2289)	Unfractionated-Heparin Group (n = 2281)	Relative Risk (95% CI)
Death, MI, urgent target-vessel revascularization, or major bleeding	190 (8.3%)	199 (8.7%)	0.94 (0.77–1.15)
Death	3 (0.1%)	4 (0.2%)	
MI	128 (5.6%)	110 (4.8%)	
Major bleeding	70 (3.1%)	104 (4.6%)	0.66 (0.49–0.90)
Urgent target-vessel revascularization	19 (0.8%)	17 (0.7%)	

Table adapted from: [30].

**Table 4:** Results of the DREAM trial.

Endpoint	Rosiglitazone Group (n = 2635)	Placebo Group (n = 2634)	HR (95%CI)	*p*
Composite primary outcome	306 (11.6%)	686 (26.0%)	0.40 (0.35–0.46)	<0.0001
Diabetes	280 (10.6%)	658 (25.0%)	0.38 (0.33–0.44)	<0.0001
Death	30 (1.1%)	33 (1.3%)	0.91 (0.55–1.49)	0.7

Table adapted from: [34].

**Table 5:** Results of the CRISTAL trial.

Endpoint	No./Total (%) Aspirin (n = 5416)	No./Total (%) Enoxaparin (n = 3787)	Estimated Treatment Difference, % (95% CI)	*p* Value
Composite primary outcome	187/5416 (3.5)	69/3787 (1.8)	1.97 (0.54 to 3.41)	0.007
Pulmonary embolism within 90 d	58/5416 (1.1)	21/3787 (0.6)	0.44 (−0.19 to 1.08)	0.17
Any deep venous thrombosis within 90 d	140/5416 (2.6)	50/3787 (1.3)	1.61 (0.54 to 2.68)	0.003
Both pulmonary embolism and deep venous thrombosis within 90 d	11/5416 (0.2)	2/3787 (0.1)	0.10 (−0.10 to −0.30)	0.32
Above-knee deep venous thrombosis within 90 d	12/5415 (0.2)	6/3787 (0.2)	0.06 (−0.11 to 0.23)	0.49
Below-knee deep venous thrombosis within 90 d	129/5415 (2.4)	45/3787 (1.2)	1.49 (0.48 to 2.50)	0.004

Table adapted from: [35].

**Table 6:** Results of the LIFE trial.

Endpoint	Losartan (n = 4605)	Atenolol (n = 4588)	Adjusted Hazard Ratio (95% CI)	*p*
Primary CEP	508 (11%)	588 (13%)	0.87 (0.77–0.98)	0.021
Cardiovascular mortality	204 (4%)	234 (5%)	0.89 (0.73–1.07)	0.206
Stroke	232 (5%)	309 (7%)	0.75 (0.63–0.89)	0.001
MI	198 (4%)	188 (4%)	1.07 (0.88–1.31)	0.491

Table adapted from: [38].

**Table 7:** Results from Webster et al. clinical trial.

Endpoint	Intervention Group (n = 379)	Control Group (n = 376)	Relative Risk (95% CI)
Catheter failure per person	143 (38%)	123 (33%)	1.15 (0.95 to 1.40)
Phlebitis	16 (4%)	12 (3%)	1.32 (0.63 to 2.76)
Infiltration	135 (36%)	120 (32%)	1.12 (0.91 to 1.36)

Table adapted from: [40].

**Table 8:** Results of the HOPE study.

Endpoint	Ramipril Group (n = 4645)	Placebo Group (n = 4652)	Relative Risk (95% CI)	*p* Value
MI, stroke, or death from cardiovascular causes	651 (14.0)	826 (17.8)	0.78 (0.70–0.86)	<0.001
Death from cardiovascular causes	282 (6.1)	377 (8.1)	0.74 (0.64–0.87)	<0.001
MI	459 (9.9)	570 (12.3)	0.80 (0.70–0.90)	<0.001
Stroke	156 (3.4)	226 (4.9)	0.68 (0.56–0.84)	<0.001

Table adapted from: [41].

**Table 9:** Practical approach to interpret CEPs.

Questions	Importance for Interpretation
Are the components of the CEP of similar importance to patients?	Similar importance of each component of the composite would allow the assumption that the effect of the intervention is comparable on each component of the composite
Did the endpoints with different importance occur with similar frequency?	If the occurrence of the components is not similar, then the CEP will be mostly determined by the predominant event
Are the component end points likely to have similar relative risk reductions?	This question allows to see if the treatment effect was similar in all components.

Table adapted from: [12].

**Table 10:** Advantages and limitations of the proposed novel statistical approaches to analyze CEPs.

Novel Statistical Method	Advantages	Limitations	
Win ratio	It considers the severity of the components of the composite More of the clinically important components are included in the analysis Calculations are easily performed	The ranking of the components is not based on universal consensus and can be debatable Lack of familiarity among triallists It only considers whether the event occurred sooner in one patient compared to the other, not the actual time from randomization to event occurrence	[54,56,57]
Negative binomial regression	Considers the total events per patient Can evaluate the effect of the treatment on recurring events	The same follow-up duration should be applied per patient, which is difficult to achieve Assigns equal weight to all components	[54]
Andersen–Gill Model	Considers the total events per patient (useful to analyze recurrent events) The risk of an event is not affected by the occurrence of a previous event	Assigns equal weight to all components	[50,58]
Weighted composite endpoint	Incorporates all events into the analysis while considering their severity	The weight assigned to each component can be debatable because a universal consensus on the severity of each event does not exist	[51,53,54,59,60]

## Data Availability

Not applicable.
